# Big Data Analysis and Prediction System Based on Improved Convolutional Neural Network

**DOI:** 10.1155/2022/4564247

**Published:** 2022-03-10

**Authors:** Xuegong Du, Xiaojun Cao, Rui Zhang

**Affiliations:** College of Information Engineering, Lanzhou University of Finance and Economics, Lanzhou 730020, China

## Abstract

This paper presents a big data analysis and prediction system based on convolutional neural networks. Continuous template matching technology is used to analyze the distributed data structure of big data, and the information fusion processing of cloud service combination big data is combined with matching related detection methods, frequent item detection, and association rule feature extraction of high-dimensional fusion data. A clustering method is adopted to realize the classification and mining of cloud service portfolio big data. The hardware equipment of the car to detect the surrounding environment is complicated, and the combination of the convolutional neural network and the camera to detect the surrounding environment has become a research hotspot. However, simply using the convolutional neural network to process the camera data to control the turning angle of the car has the problems of long training time and low accuracy. An improved convolutional neural network is proposed. The experimental results show that the accuracy of data mining by this method is 12.43% and 21.76% higher than that of traditional methods, and the number of iteration steps is shorter, indicating that the timeliness of mining is higher. This network structure can effectively improve the training speed of the network and improve the accuracy of the network. It is proven that the convolutional neural network has faster training speed and higher accuracy.

## 1. Introduction

**Figure 1:**
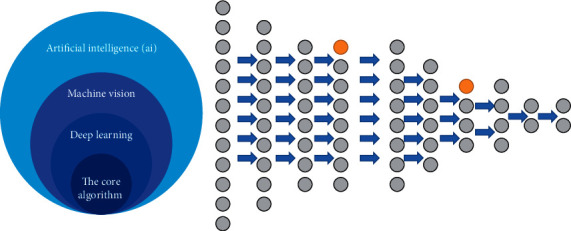
Multilayer neural network of deep learning technology.

All walks of life have begun to carry out artificial intelligence research in an all-around way, the most critical of which is the deep learning technology (Figure 1). “Deep learning” is a multilayer neural network, and “deep” in a sense refers to the number of layers of artificial neural networks. This is a brand-new field in machine learning research. This method aims to simulate human's intelligent behavior by simulating human thinking process so that, after training, the machine can show intelligent behavior that looks like a human, so that the ability of machine learning can be displayed. There is a possibility of surpassing human intelligence [1]. Deep learning has led to the rise of artificial intelligence. Traditional artificial intelligence algorithms rely on artificially summarized rules to program solutions to problems. But deep learning is different. It does not require artificial extraction of the characteristics or rules of the problem. It can learn from the input. A large amount of data spontaneously summarizes the law, adaptively adjusts its own structure so as to draw inferences from one another and generalize it to a case that has never been seen before [2]. To sum it up in one sentence, the most important feature of deep learning is that it can automatically learn W from data. Basic deep learning models can be divided into two categories: generative models and discriminative models. The former mainly includes restricted Boltzmann machine (RBN) models, autoencoder (AE) models, and deep belief network (DBN) models, which are generally used to express high levels of data. Order correlation or joint statistical distribution describing data; the latter mainly include convolutional neural network (CNN) model, recurrent neural network (RNN) model, deep stacking network (DSN) models, and long short-term memory network models, are usually used to classify the internal pattern of the data or describe the posterior distribution of the data [3].

The convolutional neural network is the most widely used deep learning network in computer vision. It has achieved good results in various image recognition tasks in various fields, such as face recognition, fingerprint recognition, license plate recognition, and target tracking [4]. However, changes in illumination and viewing angles still present certain challenges to image recognition. In order to solve this problem, Li B. proposed a color image recognition method that combines image feature data and a deep trust network to construct image color data. Under the premise of the field, wavelet transform is used to describe the multiscale features of the image, and finally, the deep trust network is trained unsupervised, but the recognition rate needs to be further improved [5]. Rao M. et al. proposed an image recognition method based on the CNN-GRNN model and designed a new image recognition model that extracts multilayer features in the image through a convolutional network and uses a generalized regression neural network to replace the reverse spread the neural network to improve the generalization ability and robustness of the classifier [6]. Clabaut, T. fused Bayesian networks and artificial neural networks to create a model. The fusion model can well reflect the correlation of road traffic flow in time and space. The study verifies the prediction results of the fusion model by using the traffic flow data collected by large floating vehicles installed on the Roman road network. The verification results show that the spatial structure of the Bayesian network is effective in road traffic flow prediction under general conditions. In a few cases of nonrecurring congestion, it is more valuable to use a single-dimensional time series method to process data [7].

On the current basis, this paper proposes a kind of information fusion processing based on the combination of cloud services and big data. First, frequent item detection and association rule feature extraction are performed on the high-dimensional fusion data. Then, they use a convolutional neural network classifier to classify the extracted association rules. Combine the feature compression method to reduce the dimensionality of the classified output cloud service portfolio big data and use the clustering method to realize the classification and mining of the cloud service portfolio big data; the designed network structure adopts unsupervised bipartite K-means and convolutional neural networks. Compared with the traditional convolutional neural network, the combined method of (CNN) can reduce the training parameters and eliminate the problem of gradient dispersion.

## 2. Distributed Structure Model and Feature Extraction of Big Data

### 2.1. Feature Distribution Model of Cloud Service Portfolio Big Data

**Figure 2:**
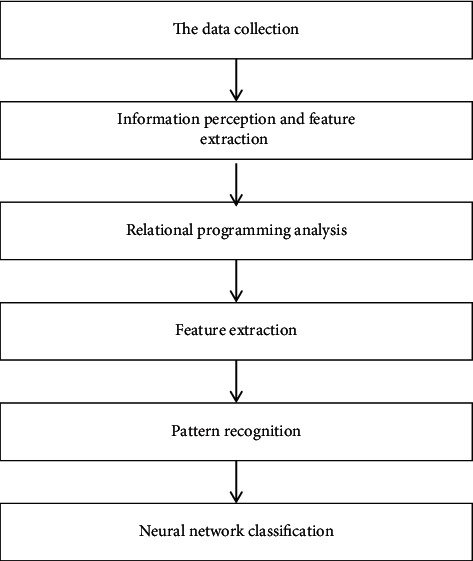
The overall structure model of cloud service portfolio big data mining.

In order to realize the optimized mining of cloud service combination big data, firstly build the distributed data structure model of big data under the cloud service combination mode and use the quadruple *G* to represent the distributed storage center of cloud service combination big data, *G*=(V, *E*, W, C) [8]. Assuming *d* is the phase space embedding dimension of the cloud service combination big data interaction, the multi-nonlinear component joint statistical method is used to reconstruct the high-dimensional feature space of the cloud service combination big data, and the clustering method is used for the adaptive classification of big data. Based on the above analysis, the overall structure model of the cloud service portfolio for big data mining is constructed, as shown in Figure 2.

There are many disturbance factors in the process of cloud service portfolio big data mining, which are time-varying and random [9]. The clustering method is used for big data information fusion, and the association rule term constraint equation is used to express the information flow model of cloud service portfolio big data, and it is expressed as

(1) $$\begin{array}{c} x_{n}=x\left(t_{0}+n\Delta t\right)=h\left[z\left(t_{0}+n\Delta t\right)\right]+\omega_{n}, \end{array}$$

where *h* is the distributed time series of cloud service combination big data, expressed as a function with a multidimensional data structure model and *ω*_*n*_ is the observation or measurement error of big data multisensor information fusion tracking. The distribution function description formula of the distribution structure model of cloud service portfolio big data is

(2) $$\begin{array}{c} X_{p}\left(u\right)=\left\{\begin{array}{cc} p\sqrt{\frac{1-j\;\cot\;a}{2\pi }}e^{j\left(u2/2\right)\cot\;a}\int_{-\infty }^{+\infty }x\left(t\right)e^{j\left(u2/2\right)\cot\;a-jtu\;\csc\;a}\text{d}t, & a\neq n\pi ,\\ x\left(u\right), & a=2n\pi ,\\ x\left(-u\right), & a=\left(2n\pm 1\right)\pi , \end{array}\right. \end{array}$$

where *p* is the order of the big data storage structure of the distributed cloud service combination and *α* is the time window width of statistical information sampling. Then we construct a temporal structure model of cloud service portfolio big data distribution.

**Figure 3:**
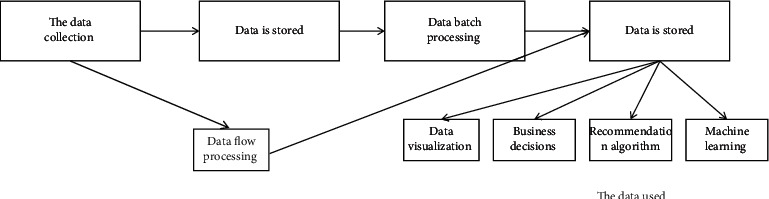
Distributed data structure of big data.

The mined cloud service combination big data is reconstructed according to the five-tuple of the association rule item characteristics, the association rule knowledge base is constructed, and the characteristic identification function of the cloud service combination big data data structure is given as *P*_*c*_=∑_*i*=0_^*n*^∑_*j*=0_^*n*^*a*(*i*, *j*)*P*(*i*, *j*). The statistical regression analysis method is used to construct the nonlinear time series model of the cloud service combination big data, and the linear combination model is obtained as

(3) $$\begin{array}{c} x_{k}=\sum_{n=0}^{\left(N/2\right)-1}2\left(a_{n}\cos\frac{2\pi kn}{N}-b_{n}\sin\frac{2\pi kn}{N}\right),\;k=0,1,\ldots ,N-1, \end{array}$$

where *a*_*n*_ represents the magnitude of the cloud service portfolio big data linear programming model. For a set of continuous cloud service combination big data, the continuous template matching technology is used to analyze the distributed data structure of big data, and the information fusion processing of cloud service combination big data is combined with matching-related detection methods. The data flow processing is shown in Figure 3.

### 2.2. Data Feature Extraction

Suppose the amount of cloud service combination big data nodes is *m*, and the closed frequent itemset feature extraction output of each node is expressed. Based on the extreme learning method for the global optimization of data feature extraction, the mathematical expression of the linear programming problem for constructing cloud service portfolio big data mining is as follows:

(4) $$\begin{array}{c} \min\left(f\right)=\sum_{i=1}^{m}\sum_{j=1}^{n}C_{ij}X_{ij},\\ \text{s.t }\left\{\begin{array}{cc} \sum_{j=1}^{m}X_{ij}=a_{i}, & i=1,2,\ldots ,m,\\ \sum_{i=1}^{m}X_{ij}=b_{i}, & j=1,2,\ldots ,n,\\ X_{ij}\geq 0, & i=1,2,\ldots ,m;j=1,2,\ldots ,n. \end{array}\right. \end{array}$$

Assuming that the current number of cloud service portfolio big data distribution nodes is *n*, *N*_1*n*_,…, *N*_*n*_, the load of the big data to be mined in the link layer is *L*_1_,…, *L*_*n*_, and the estimated characteristics of the cloud service portfolio big data mining output are *P*_1_^min^,…, *P*_*n*_^min^ [10]. In the linear programming model, the feature decomposition of cloud service portfolio big data is carried out, wavelet entropy is obtained, and the information fusion processing of cloud service portfolio big data is combined with matching correlation detection methods. Perform frequent item detection on high-dimensional fusion data to realize the feature extraction of association rules, which is expressed as

(5) $$\begin{array}{c} \left\{\begin{array}{c} \rho_{t}^{I}=\frac{\sum_{1}^{N_{I}}s\left(i,t\right)}{V}=\frac{N_{I}}{V},\\ \rho_{t}^{R}=\frac{\left|\sum_{1}^{N_{R}}s\left(i,t\right)\right|}{V}=\frac{N_{R}}{V},\\ \rho_{t}^{s}=\frac{N_{S}}{V}. \end{array}\right. \end{array}$$

Among them, *N*_1_, *N*_*R*_, and *N*_*s*_, respectively, represent the average mutual information feature quantity and state distribution set of cloud service portfolio big data [11].

### 2.3. Data Classification and Mining Technology Optimization

#### 2.3.1. Convolutional Neural Network Classification

Based on the abovementioned large data distributed data structure analysis and association rule feature extraction using continuous template matching technology, the optimization design of data classification mining algorithms is carried out. This paper proposes a big data classification mining technology based on convolutional neural networks, using statistical average methods to build a regression analysis model of the big data that needs to be mined and it is expressed as

(6) $$\begin{array}{c} \left\{\begin{array}{c} \min\sum_{1\leq i\leq Ke\subseteq k\left(e\right)}\sum \frac{f\left(e\left(i\right)\right)}{C\left(e,i\right)},\\ \begin{array}{c} 0\leq f\left(e,i\right)\leq C\left(e,i\right),\\ F=\text{const,} \end{array}\\ \sum_{1\leq i\leq K,e\subseteq k\left(e\right)}\frac{f\left(e\left(i\right)\right)}{C\left(e,i\right)}+\sum_{e\subseteq k\left(e\right)}\frac{f\left(e^{\prime }\left(i\right)\right)}{C\left(e^{\prime },i\right)}\leq k\left(v\right). \end{array}\right. \end{array}$$

A multivariate statistical characteristic equation is used to describe the fitting state model of cloud service portfolio big data as

(7) $$\begin{array}{c} \left(\begin{array}{c} X\\ P\left(X\right) \end{array}\right)=\left\{\begin{array}{c} a_{1},a_{2},\ldots ,a_{m}\\ p\left(a_{1}\right),p\left(a_{2}\right),\ldots ,p\left(a_{m}\right) \end{array}\right\}. \end{array}$$

Among them, 0 ≤ *p*(*a*_*i*_) ≤ 1 (*i*=0,1,2,…, *m*) and ∑_*i*=1_^*m*^*p*(*a*_*i*_)=1 represent the autoregressive statistical characteristic parameters of cloud service portfolio big data. A convolutional neural network classifier is used to classify the attributes of the extracted association rules of cloud services combined with big data [12]. The convolutional neural network is a three-layer network structure, and the input and output iteration equation of the convolutional neural network classifier is

(8) $$\begin{array}{c} W\left(n+1\right)=W\left(n\right)-\eta \frac{\partial E}{\partial W}+\partial \Delta \;W\left(n\right). \end{array}$$

Assuming that the learning step size of the convolutional neural network for big data recognition is

(9) $$\begin{array}{c} w_{sij}\left(n_{0}+1\right)=w_{sij}\left(n_{0}\right)-\eta_{sij}\frac{\partial J}{\partial w_{sij}}. \end{array}$$

Using the learning algorithm of the convolutional neural network, the adaptive learning weighting coefficient of the cloud service portfolio big data classification is obtained as

(10) $$\begin{array}{c} a_{\text{desira}}^{i}=a_{1}\cdot \frac{{\text{Density}}_{i}}{\sum_{i}{\text{Density}}_{i}}+a_{2}\frac{{\text{AP}}_{i}}{{\text{AP}}_{\text{init}}}. \end{array}$$

Under the constraints of *B*⇒*D*, *A*∩*B*⇒*D*, and other rules, the attribute set of cloud service portfolio big data classification satisfies

(11) $$\begin{array}{c} \left\{\begin{array}{c} a_{1}+a_{2}=1,\;a_{1},a_{2}\in \left[0,1\right],\\ a_{2}=\frac{\max_{i}\left({\text{AP}}_{i}\right)-\min_{i}\left({\text{AP}}_{i}\right)}{{\text{AP}}_{\text{init}}}. \end{array}\right. \end{array}$$

**Figure 4:**
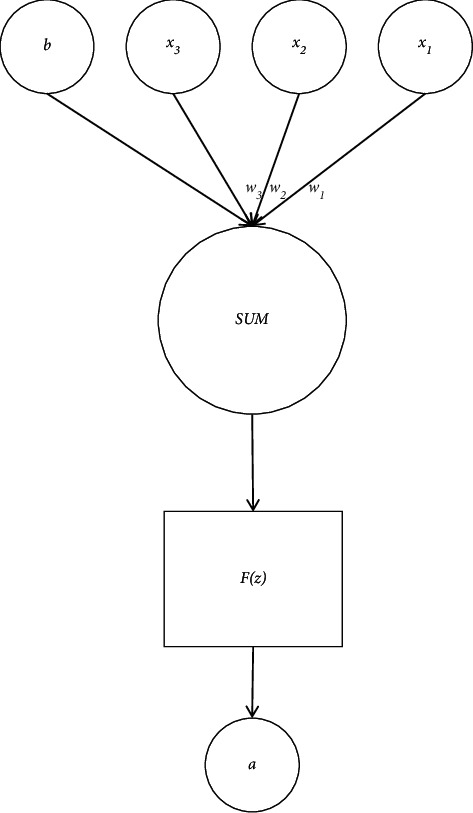
Convolutional neural network neuron structure.

The statistical quantitative set of data is (*u*, *v*) ∈ *E*, suppose *A* ⊂ *V*, *B* ⊂ *V*, and *A*∩*B*=*ϕ*. The convolutional neural network classifier is used for attribute classification to realize the big data reorganization and data structure rearrangement of the cloud service portfolio. The neuron structure of the convolutional neural network is shown in Figure 4.

#### 2.3.2. Data Feature Dimensionality Reduction and Classification Mining Output

Based on the use of convolutional neural network classifiers for attribute classification, in order to reduce the computational cost, combined with the feature compression method, the dimensionality reduction processing of the cloud service composite big data output by the classification is performed [13]. The feature compressor is described as

(12) $$\begin{array}{c} F=\bar{p}\left(x,y\right)=p\left(x,y\right){\left(\frac{v\left(x\right)}{v\left(y\right)}\right)}^{1/2}. \end{array}$$

Among them

(13) $$\begin{array}{c} p\left(x,y\right)=\frac{k\left(x,y\right)}{v\left(x\right)},\\ v\left(x\right)=\sum_{y}k\left(x,y\right). \end{array}$$

The conceptual grid node of cloud service portfolio big data obtained by using a convolutional neural network classifier is

(14) $$\begin{array}{c} X^{\prime }={\left(x_{i0}^{\prime },x_{i1}^{\prime },\ldots ,x_{i\left(n-1\right)}^{\prime },y_{i0}^{\prime },y_{i1}^{\prime },\ldots ,y_{i\left(n-1\right)}^{\prime }\right)}^{T}. \end{array}$$

The dimensionality reduction of the cloud service portfolio big data output by classification is performed, and the clustering method is used to realize the classification mining of the cloud service portfolio big data [14].

## 3. Method for Predicting Lateral Turning Angle of Trolley Based on Bipartite K-Means Convolutional Neural Network

### 3.1. Trolley Lateral Control

**Figure 5:**
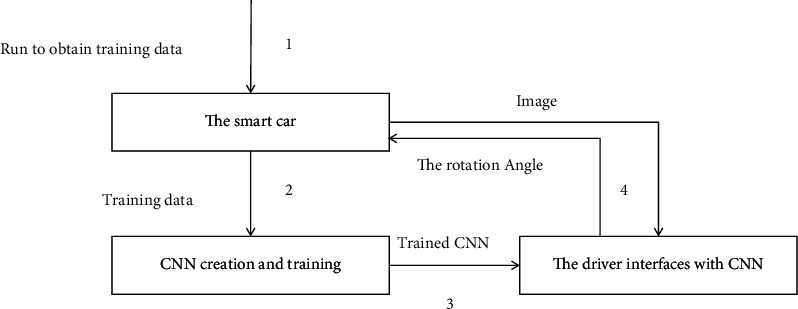
Overall flow chart.

The lateral control of the trolley is divided into image acquisition, image preprocessing, CNN model establishment, and prediction angle in this article. The overall process is shown in Figure 5.

### 3.2. Building a Dataset

**Figure 6:**
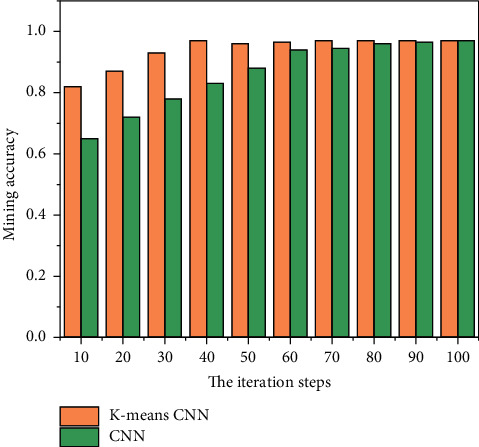
Comparison of data mining accuracy.

A camera is preinstalled on the front, left, and right parts of the front end of the trolley, and then the trolley is manually controlled to avoid obstacles, and the image data of the three cameras are obtained from the upper computer. The data of the three cameras are divided into three parts: front, left, and right. At the same time, the steering gear angle is converted into a digital signal through an adjustable resistor, and then the digital signal is converted into the rotation angle of the front wheel of the trolley by the host computer [15]. In the process of data collection, in order to ensure the validity of the dataset in a variety of complex situations, the data collection should meet the following requirements:

1. In order to avoid the influence of light, weather, and other factors on the camera, the dataset should be collected under different light and weather conditions in the same environment.
2. In order to avoid the occurrence of too many abnormal quantities, we try to keep the angle similar to the original under the same environment, and try to avoid the appearance of the angle that deviates too much from the original [16]. When the above conditions are met, the article collected more than 400 images at each of the front, left, and right positions of the trolley. The comparison of the accuracy of data mining is shown in Figure 6.

### 3.3. Dataset Preprocessing

**Figure 7:**
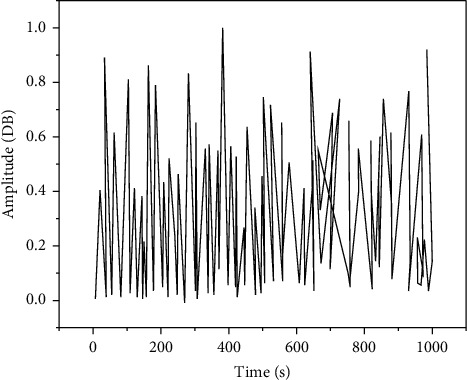
Time-domain waveform of data mining.

In order to ensure that the number of images is sufficient to prevent the occurrence of overfitting, this article amplifies the dataset by adding Gaussian noise and salt-and-pepper noise to the original image, and finally obtains more than 1,400 images in each of 3 locations, totaling more than 4200 image dataset, the time-domain waveform of data mining is shown in Figure 7.

**Figure 8:**
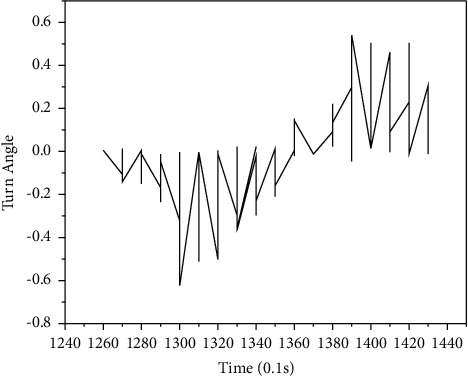
Comparison of the original rotation angle and the smoothed rotation angle.

Since the turning angle is converted by a digital signal, the change of the turning angle in time is not smooth. In order to smooth the data, this article selects a moving average and calculates the average with 3 time units (0.3 s) as a window.  Figure 8 is a comparison diagram of the original data and the smoothed data over time.

### 3.4. Establishment of Convolutional Neural Network Model Based on Bipartite K-Means

#### 3.4.1. Network Structure

This section explains the improved convolutional neural network structure for the prediction system of the lateral turning angle of the trolley. There are 1 K-means layer, 3 ordinary convolutional layers, 2 fully connected layers, and 2 dropout layers [17]. Among them, 3 convolutional layers and 2 fully connected layers are trainable layers. The height and width of the convolutional layer decrease as the depth of the network structure increases. The output node of the fully connected layer of the last layer is 1. In front of the fully connected layer, add a dropout layer to improve the generalization ability of the network and prevent overfitting [18].

#### 3.4.2. Input Layer

The preprocessed image pixels are 160 × 320, and the preprocessed images at the front, left, and right positions are used as the input of the input layer. The purpose is to preserve the image information of each position of the vehicle as much as possible.

#### 3.4.3. Dichotomous K-Means Layer

When traditional convolutional neural networks are used in image recognition, a huge dataset is required for training, and it is prone to overfitting problems. Therefore, it is thought of clustering the pictures first. The classic K-means clustering algorithm is greatly affected by the initial clustering center and it is easy to converge to the local minimum, so the bipartite K-means algorithm is used [19]. Using the bisection K-means algorithm can reduce the amount of training overhead, overcome the local optimal problem caused by the uneven distribution of the dataset, and the clustering effect is better. The images at the front, left, and right positions are passed through the bisected K-means layer in turn, and the *k* clustering results obtained are used as the input of the next layer. The *k* value of the dichotomous K-means layer designed in this paper is set as 3.

#### 3.4.4. Convolutional Layer

**Figure 9:**
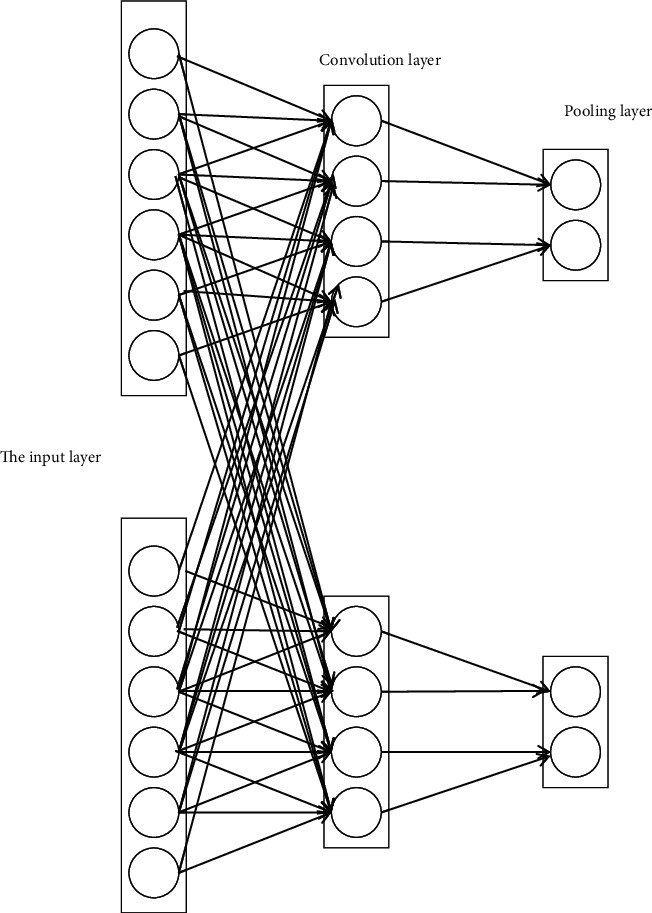
K-means convolutional neural network model.

The convolutional layer extracts the characteristics of the input image through convolution calculation. The network structure designed in this paper has 3 convolutional layers, and each convolutional layer decreases in turn as the network depth increases. The first convolutional layer has 16 filters, and the size of each filter is 8 × 8 × 3, and the step size is 4; the second convolutional layer has 32 filters, and the size of each filter is 5 × 5 × 3, and the step size is 2; the third convolutional layer has 64 filters, each filter has a size of 5 × 5 × 2, and the step size is 2. There are many types of activation functions in convolution calculations. Among them, the sigmoid function is the most commonly used, and it can also be a modified linear unit (ReLu). The modified linear unit used here is to speed up the training of the network and reduce the calculation time of the network (Figure 9).

(15) $$\begin{array}{c} f\left(x\right)=\max\left(0,\underset{i}{\sum }w_{i}a_{i}\right). \end{array}$$

In the formula *w* is the connection weight and *a* is the output of the previous layer.

#### 3.4.5. Output Layer

**Figure 10:**
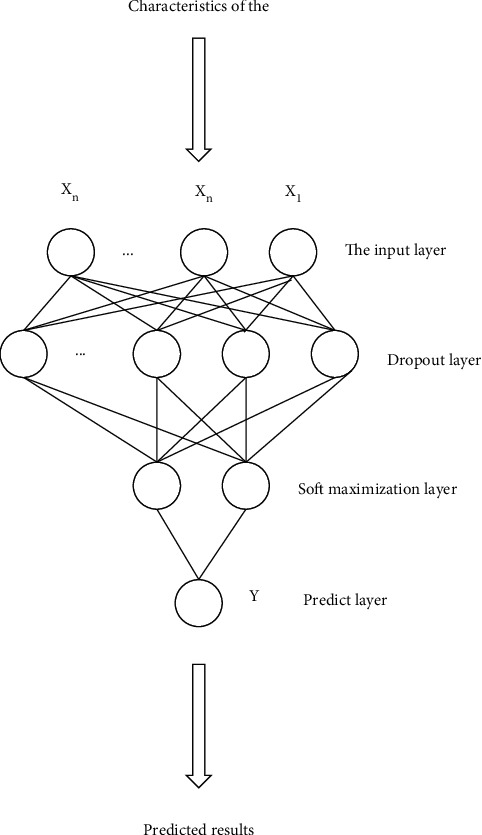
Classification layer network structure.

The output layer of the network structure is a fully connected layer, and the output node of the fully connected layer is 1. In order to avoid overfitting, a dropout layer is added in front of the fully connected layer, and the dropout rate of the dropout layer is 0.5. A dropout layer with a dropout rate of 0.2 is also added to another fully connected layer of the network structure. The network structure of the classification layer is shown in Figure 10.

## 4. Experimental Results and Analysis

**Figure 11:**
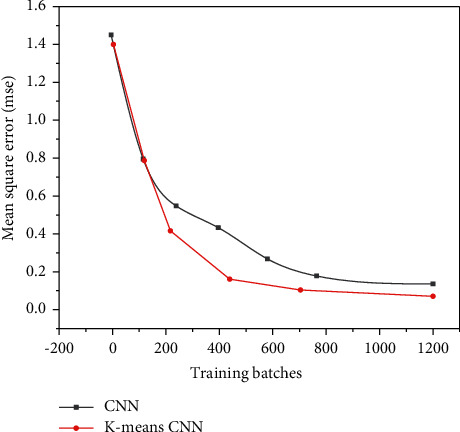
The first comparison of results based on the bipartite K-means convolutional neural network and the traditional convolutional neural network.

**Figure 12:**
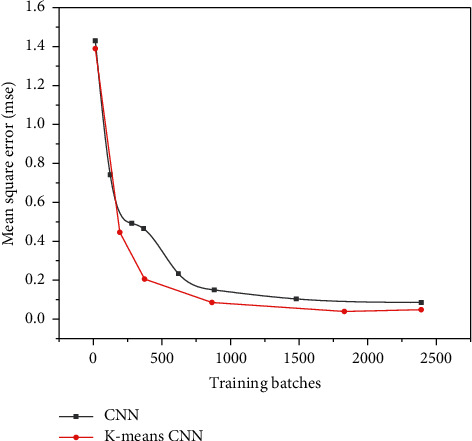
The second result comparison between the bipartite K-means convolutional neural network and the traditional convolutional neural network.

**Figure 13:**
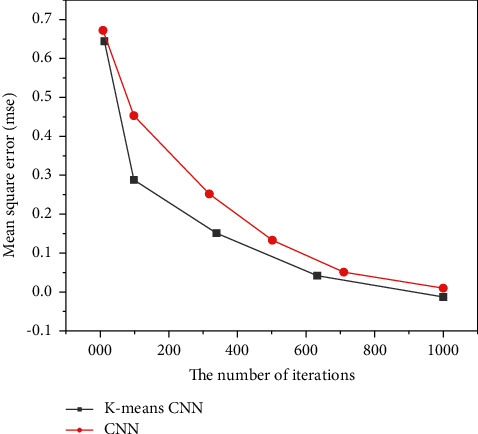
Comparison of training results based on the bipartite K-means convolutional neural network and the traditional convolutional neural network.

**Figure 14:**
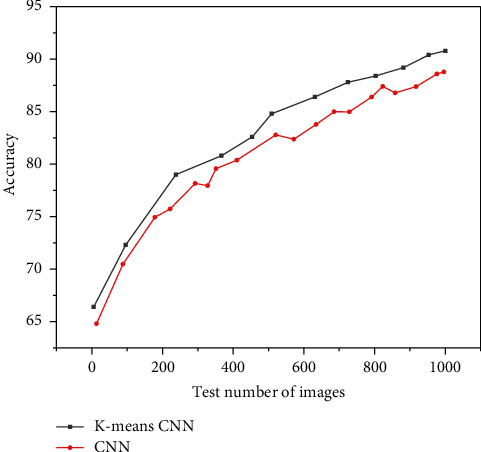
Comparison of accuracy based on the bipartite K-means convolutional neural network and the traditional convolutional neural network.

**Figure 15:**
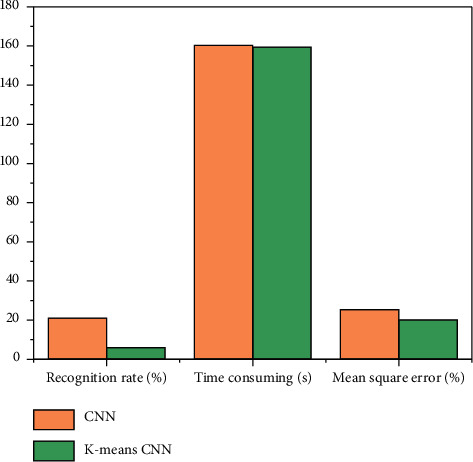
Error comparison between the bipartite K-means convolutional neural network and the traditional convolutional neural network.

The training results of the convolutional neural network based on dichotomous K-means and the traditional convolutional neural network are shown in Figures 11–13, and the comparison results of accuracy and error results are shown in Figures 14 and 15.

It can be seen from Figure 13 that although the two networks eventually tend to converge, if only the convolutional neural network is used for angle prediction, more iterations are needed to gradually converge. If a bipartite K-means convolutional network is added, the image category can be determined faster, so that fewer iterations are required.

It can be seen from Figures 14 and 15 that the recognition rate of the two network structures increases with the increase of the number of test pictures.

## 5. Conclusion

A convolutional neural network classifier is used for attribute classification, combined with a feature compression method to reduce the dimensionality of the cloud service portfolio big data output by classification, and a fuzzy clustering method is used to realize the classification and mining of cloud service portfolio big data. The bisection K-means clustering method is added to the traditional convolutional neural network for optimization, and it is verified on the image data collected by the smart car camera. The improved convolutional neural network uses the binary K-means clustering method to perform clustering learning first, so that the convolutional neural network can obtain richer input information, thereby reducing the training parameters of the convolutional neural network, accelerating the training speed of the network, and improving training accuracy. The experimental results show that the improved convolutional neural network model has faster training speed and higher accuracy than the traditional convolutional neural network.

## Data Availability

The data used to support the findings of this study are available from the corresponding author upon request.
